# Hydrogen Gas Inhalation Treatment for Coronary Artery Lesions in a Kawasaki Disease Mouse Model

**DOI:** 10.3390/life14070796

**Published:** 2024-06-24

**Authors:** Wen-Ling Shih, Tsung-Ming Yeh, Kuang-Den Chen, Steve Leu, Shih-Feng Liu, Ying-Hsien Huang, Ho-Chang Kuo

**Affiliations:** 1 Department of Biological Science and Technology, National Pingtung University of Science and Technology, Neipu 912301, Taiwan; wlshih@mail.npust.edu.tw (W.-L.S.); ytm@mail.npust.edu.tw (T.-M.Y.); 2 General Research Service Center, National Pingtung University of Science and Technology, Neipu 912301, Taiwan; 3 Institute for Translational Research in Biomedicine, Kaohsiung Chang Gung Memorial Hospital, Kaohsiung 83301, Taiwan; dennis8857@gmail.com (K.-D.C.); st.leu.tw@gmail.com (S.L.); 4 Kawasaki Disease Center, Department of Pediatrics, Kaohsiung Chang Gung Memorial Hospital, Kaohsiung 83301, Taiwan; 5 Department of Biotechnology, College of Life Science, Kaohsiung Medical University, Kaohsiung 83301, Taiwan; 6 Department of Respiratory Therapy, Kaohsiung Chang Gung Memorial Hospital, Kaohsiung 83301, Taiwan; liuphysico@yahoo.com.tw; 7 College of Medicine, Chang Gung University, Taoyuan 33302, Taiwan

**Keywords:** Kawasaki disease, animal model, hydrogen gas

## Abstract

Background: Kawasaki disease (KD) is a syndrome primarily affecting young children, typically under the age of five, and is characterized by the development of acute vasculitis. Through extensive research conducted on both murine and human subjects, it has been demonstrated that heightened levels of reactive oxygen species (ROS) play a pivotal role in the development of KD, especial coronary artery lesions (CALs). Hydrogen gas exhibits potent antioxidant properties that effectively regulate ROS production and the inflammatory response. Methods: We used *Lactobacillus casei* cell wall extract (LCWE)-induced vasculitis in mice as an animal model of KD and treated the mice with hydrogen gas inhalation. Results: We observed significant dilatation and higher Z scores in the left coronary artery (LCA) in D21 and D28 in mice after LCWE treatment compared to the control group (*p* < 0.001) and a significant resolution of LCA diameters (*p* < 0.01) and Z scores (*p* < 0.01) after treatment with inhaled hydrogen gas. We further demonstrated that serum IL-6 expression was higher in mice after LCWE treatment (*p* < 0.01) and IL-6 significantly decreased after inhaled hydrogen gas therapy (*p* < 0.001). Conclusion: According to our literature review, this is the first report where hydrogen gas inhalation has been demonstrated to be effective for the treatment of coronary artery dilatation in a KD murine model.

## 1. Introduction

Kawasaki disease (KD) is a syndrome characterized by acute vasculitis that impacts children, mostly under the age of five, who have a genetic predisposition [1,2]. In 1974, Dr. Tomisaku Kawasaki published a report in English describing 50 cases of KD [3]. This syndrome is characterized by prolonged fever, conjunctivitis, inflammation of the mucous membranes, various types of skin rashes, swelling and hardening of the hands and feet with peeling fingertips, and swelling of cervical lymph nodes, predominantly on one side [1]. Coronary artery lesions (CALs) are the most severe complication of KD, and can result in myocardial infarction and the development of coronary artery aneurysms (CAAs) [1]. If left untreated, approximately 20% of affected children may experience a sequel of vasculitis with the formation of CAAs. This can lead to symptoms such as chest pain, myocardial infarction, and even sudden cardiac death within the community [2,4]. Although we have made significant progress in comprehending KD, there is still a need for further enhancements in our understanding of its intricate mechanisms [5].

The incidence of KD varies widely among different nations. It is highest in North-East Asian countries, with almost 1 in 100 children in Japan affected by the disease by the age of 5. In contrast, the lowest incidence is reported in sub-Saharan Africa. The etiology of KD is still uncertain; it is hypothesized to result from an interaction between genetic predisposition and various environmental and immunological factors. Several susceptibility genes have been identified that are associated with the development of KD and an increased risk of coronary artery lesions. Coronary artery lesions, including transient dilatation, will be found in about 20–30% of KD patients even after intravenous immunoglobulin (IVIG) treatment. This highlights the low availability of effective treatments against this threatening condition and underscores the need for more investment in discovering new therapies.

Mitochondria play a vital role in sustaining life by regulating energy generation, facilitating signal transduction, and contributing to the production of reactive oxygen species (ROS). In recent studies, mitochondria have been recognized as crucial catalysts in the activation of inflammation, which is closely linked to processes such as apoptosis and cell death [6,7]. Hydrogen gas, known for being the lightest and most odorless molecule in the environment, exhibits potent antioxidant properties that effectively regulate ROS production and the inflammatory response [8]. The inhalation of hydrogen gas has demonstrated effective enhancement of heart function in rat models of acute myocardial infarction [9], and this improvement is achieved by regulating ROS and mitigating NLRP3 inflammasome-mediated pyroptosis. Wang et al. [10] reported that elevated NOx (NO3- + NO2-) levels in patients with KD were significantly associated with the occurrence of coronary artery dilation. These elevated NOx levels significantly decreased after IVIG treatment. Extensive research conducted on both murine and human subjects indicates that increased ROS plays a central role in driving the pathophysiology of KD [11], and the inhibition of NLRP3 is a potential treatment target for KD [12,13]. 

Due to the limited availability of coronary artery samples from children with acute KD, researchers utilize various animal models, such as mice, rabbits, swine, and dogs, to investigate the immunopathogenesis of the disease. These animal models serve as valuable resources for studying and exploring the mechanisms underlying KD [14]. The utilization of these animal models in KD research enables us to enhance our comprehension of the disease’s pathogenesis. By studying these models, we can investigate the immune-molecular mechanisms that contribute to vasculitis complications. This deeper understanding paves the way for the development of innovative therapeutic approaches in KD. *Lactobacillus casei* cell wall extract (LCWE) is the most commonly used to induce KD in animal models, causing murine coronary arteritis similar to CALs associated with human KD [14,15]. The presence of neutrophil and mononuclear cell infiltration in the perivascular/adventitial region of the myocardium, aortic valve or pericardium, aorta, and coronary artery is commonly observed in arteritis. This inflammation typically occurs between days 7 and 14 following the onset of the condition [13]. In the current study, we used LCWE-induced vasculitis in mice as an animal model of KD and treated the mice with hydrogen gas inhalation to develop therapeutic strategies for KD.

## 2. Methods

### 2.1. Ethics Statement and Animal Protocol

Our animal protocol was reviewed and approved by the Institutional Animal Care and Use Committee (IACUC) of Chang Gung Memorial Hospital (IRB No. 2021082406 and 2022081801), as in our previous report [15]. All methods were performed in accordance with the relevant guidelines and regulations. Four-week-old male C57BL/6NCrlBltw mice were purchased from Bio-LASCO Taiwan Co., Ltd., Taipei, Taiwan. All animals were housed in a pathogen-free barrier facility accredited by the Association for Assessment and Accreditation of Laboratory Animal Care (AAALAC) at 22 °C, with a relative humidity of 55%, in a 12 h light/12 h dark cycle, with food and sterile tap water available ad libitum. Inhalation anesthesia with isoflurane was used in this study. The study was conducted in accordance with ARRIVE guidelines.

### 2.2. LCWE-Induced KD Vasculitis Murine Model

*Lactobacillus casei* (ATCC 11578) cell wall extract (LCWE) was prepared as previously described in our laboratory [16]. Male mice at 5 weeks old were injected intraperitoneally with LCWE (2 mg/kg) for 7 continuous days (total 280 μg/mice) or PBS. Depending on the experimental design, mice were euthanized 28 days after injection. Serum was collected for further analysis. 

### 2.3. Hydrogen Gas Inhalation

**Figure 1:**
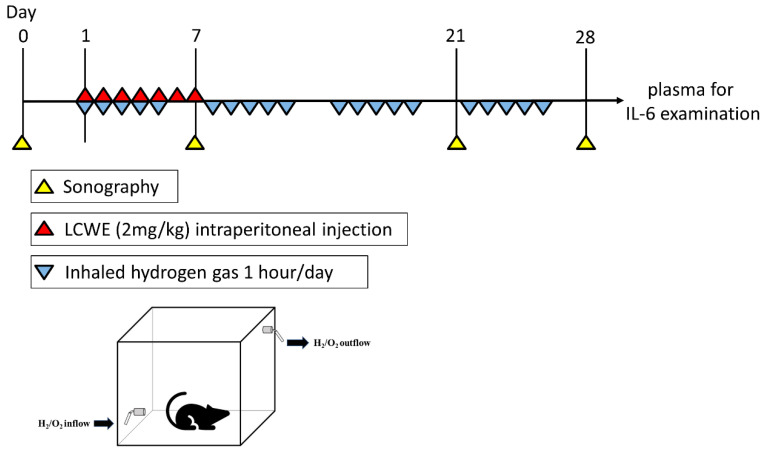
Schematic diagram of establishing an LCWE-injected mouse model followed by treatment with inhaled hydrogen gas.

We used hydrogen and an oxygen mixture gas (73% hydrogen mixed with 27% oxygen) produced using deionized water by electrolysis with a hydrogen/oxygen generator which was specifically designed to extract hydrogen and oxygen from water (model HB-133; Ota Hydrogen Biotech Co., Ltd., Kaohsiung, Taiwan). Hydrogen gas was directly introduced into the inhalation box (20× 18 × 15 cm, length × width × height) at a rate of 70–75 L/h. The box was flushed with mixed gases for 30 min to replace the air in the box. During each experiment, the concentration of hydrogen gas in the box was monitored by Thermal trace GC ultra-gas chromatography (Thermo Fisher, Waltham, MA, USA). Figure 1 shows a schematic diagram of the LCWE murine model of KD vasculitis and the therapeutics of inhaled hydrogen gas.

### 2.4. Echocardiographic and Z Score

All high-resolution echocardiography detection of mouse hearts was performed by the same senior technician. Animal echocardiography was performed by measuring the coronary artery dimensions on days 0, 7, 21, and 28 (high-resolution small-animal ultrasound [Vevo 3100, FUJIFILM VisualSonics Inc., Toronto, ON, Canada]). Z score was calculated with mean and standard deviation from data collection at day 0.

### 2.5. Measurement of Interleukin-6 Using Enzyme-Linked Immunoassay

Blood samples were collected in heparin tubes from heart chambers. The use of enzyme-linked immunoassay (ELISA) kits used for mouse plasma interleukin (IL)-6 (Catalog # KMC0061, Invitrogen, Vienna, Austria) and the methodology and assessment of performance characteristics were carried out according to the instructions.

### 2.6. Statistical Analysis

The experimental results are presented as mean ± standard error of the mean (SEM). To compare the two groups, a two-tailed Student’s t-test was employed. For determining statistical significance among multiple comparisons, a one-way analysis of variance (ANOVA) was utilized, followed by nonparametric post hoc Tukey’s t-tests and a Kruskal–Wallis test. The threshold for significance was set at *p* ≤ 0.05.

## 3. Results

### 3.1. Resolution of Dilatation of CALs in LCWE-Injected Mouse Model Following Treatment with Inhaled Hydrogen Gas

**Figure 2:**
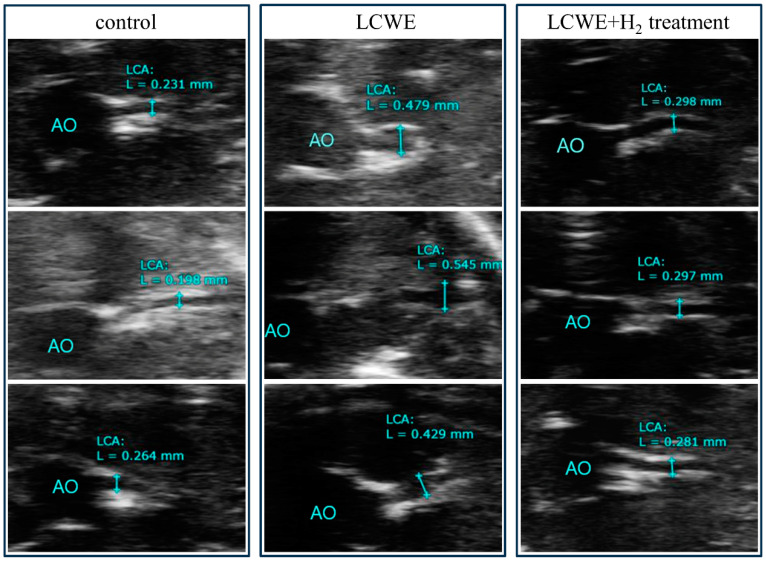
Sonography of left coronary arteries (LCAs) in the LCWE-injected mice and following inhaled hydrogen gas treatment (the measurements were made on day 21, blue lines showed the diameter of left main coronary artery).

**Figure 3:**
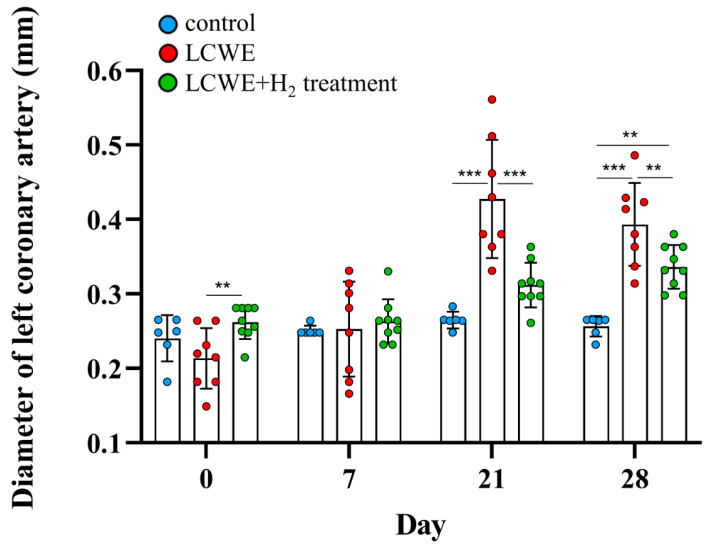
Diameter of left coronary arteries (LCA) in LCWE-injected mice. Resolution of dilatation of LCAs in mouse model injected with LCWE after treatment with inhaled hydrogen gas. Data collected from six to nine mice in each group are expressed as mean ± SE. ** *p* < 0.01 and *** *p* < 0.001 between the indicated groups.

**Figure 4:**
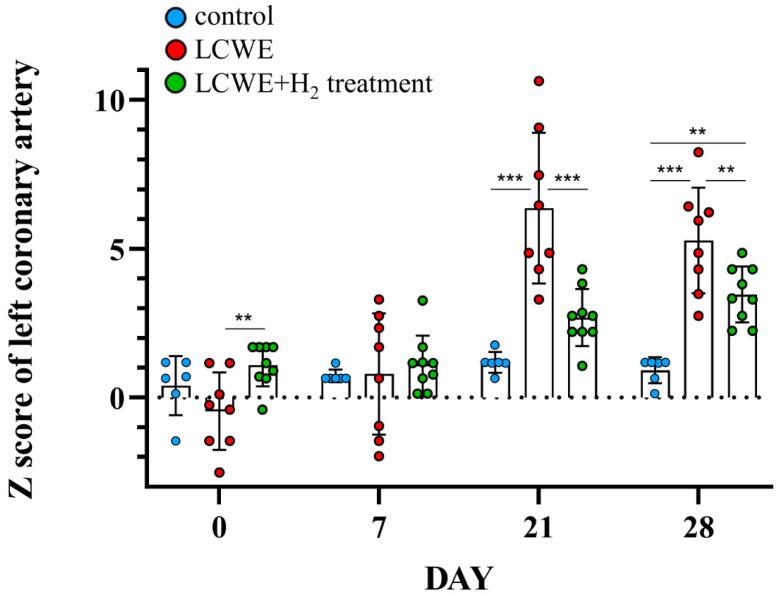
Z scores of left coronary arteries (LCAs) in LCWE-injected mice. Resolution of dilatation of LCAs in mouse model injected with LCWE after treatment with inhaled hydrogen gas. Data collected from six to nine mice in each group are expressed as mean ± SE. ** *p* < 0.01 and *** *p* < 0.001 between the indicated groups.

To gain insight into the possible involvement of inhaled hydrogen gas in preventing the development of CALs, we used an LCWE-injected mouse model, which has been shown to induce CAL formation. First, we examined the standard diameter of left coronary arteries (LCAs) from 55 five-week-old male mice (mean ± standard deviation = 0.22 ± 0.031 mm). We observed the dilation of LCAs in mice after LCWE treatment via sonography (0.43 ± 0.03 mm, N = 8, Figure 2, day 21). We next assessed the different time points between groups, and we observed significant dilatation of the LCAs on day 21 (0.43 ± 0.03 vs. 0.26 ± 0.001 mm) and day 28 (0.39 ± 0.02 vs. 0.26 ± 0.01 mm) in mice after LCWE treatment compared to the control group (N = 6) (all *p* < 0.001, Figure 3). We next examined the changes in LCA diameter after the LCWE-injected mice inhaled the hydrogen gas (N = 9). We found a significant resolution of LCA diameter after treatment with inhaled hydrogen gas (0.31 ± 0.01 vs. 0.43 ± 0.03 mm, *p* < 0.001 on day 21, and 0.34 ± 0.01 vs. 0.39 ± 0.02 mm, *p* = 0.006 on day 28, Figure 3). After normalizing the LCAs using the Z score method, we further observed increased Z scores for LCAs in LCWE-injected mice (6.37 ± 0.90 vs. 1.18 ± 0.14, *p* < 0.001 on day 21) when compared with the controls, and a significant resolution of LCA Z scores after treatment with inhaled hydrogen gas (6.37 ± 0.90 vs. 2.69 ± 0.32, *p* < 0.001 on day 21, Figure 4). 

### 3.2. Plasma Interleukin 6 in the LCWE Murine Model of KD Vasculitis

**Figure 5:**
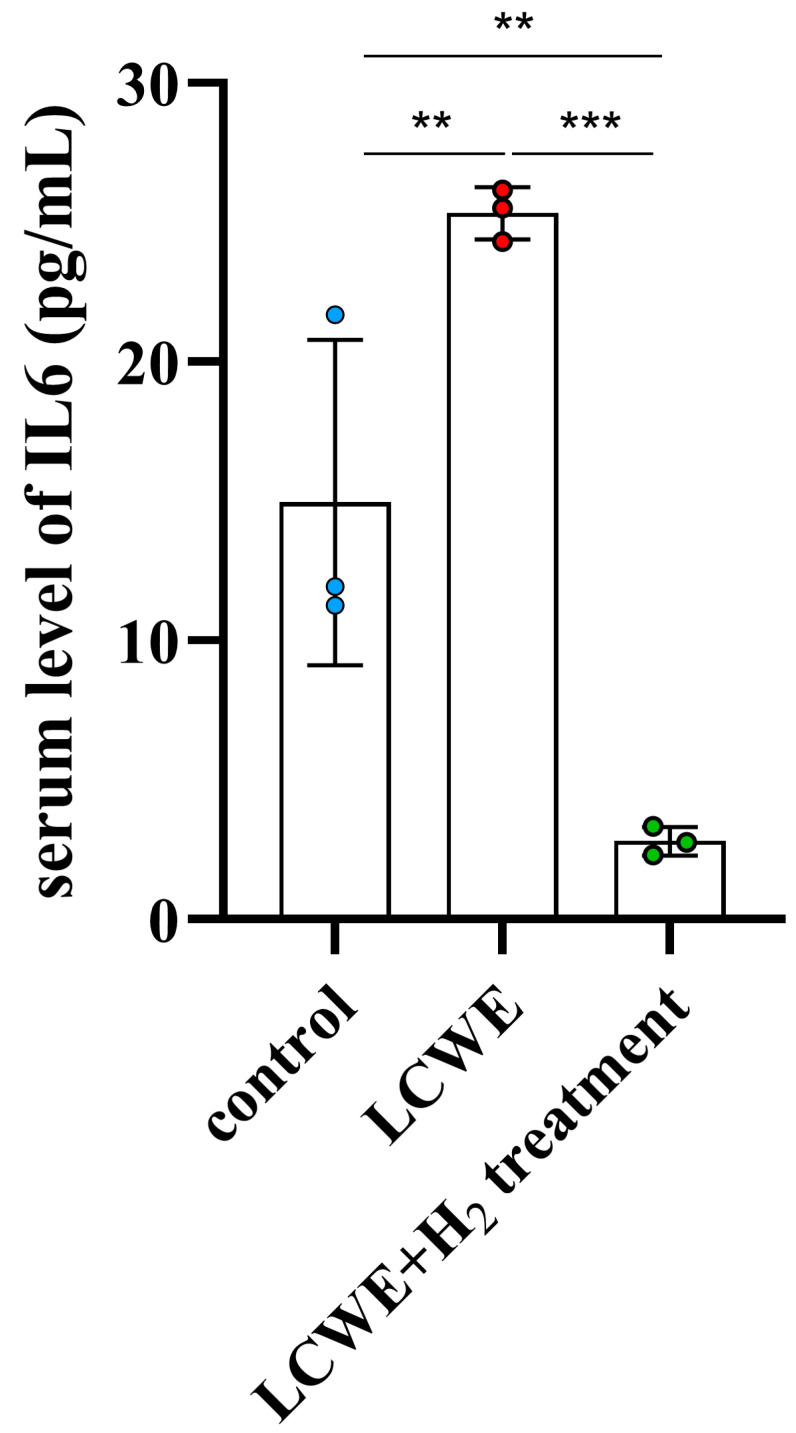
IL-6 expression in LCWE-injected mice shows a significant increase when compared with control (blue dots) (*p* < 0.01). There is a notable decrease in IL-6 expression in mouse model injected with LCWE (red dots) after treatment with inhaled hydrogen gas (green dots) (*p* < 0.001). Data collected from three mice in each group are expressed as mean ± SE. ** *p* < 0.01 and *** *p* < 0.001 between the indicated groups.

IL-6 has been implicated in the pathogenesis of KD, and elevated levels of IL-6 have been observed in the serum and affected tissues of KD patients [17]. We next determined if there are expression changes in IL-6 following LCWE-induced KD vasculitis. We observed that there was markedly induced IL-6 expression in LCWE-induced mice (*p* = 0.01) Figure 5). We also showed that inhaled hydrogen gas therapy leads to a notable decrease in IL-6 expression, which is compatible with the decrease in the formation of CALs in the LCWE murine model of KD vasculitis (*p* < 0.001, Figure 5). Overall, our data indicate that inhaled hydrogen gas therapy reduces the LCWE-induced dilatation of LCA and inflammatory cytokine IL-6 expression. 

## 4. Discussion

KD is a rare condition that primarily affects children under the age of 5. It involves inflammation of the blood vessels, particularly the small to medium-sized arteries, throughout the body. One of the most serious complications of KD is the development of CALs, which are abnormalities in the coronary arteries that supply blood to the heart. CALs can vary in severity, ranging from mild dilation or swelling of the coronary arteries to the formation of aneurysms or even coronary artery obstruction. These abnormalities can increase the risk of serious heart problems, including coronary artery disease, myocardial infarction, and in severe cases, sudden death. The early detection and treatment of KD are essential in preventing or minimizing the risk of CALs. High-dose IVIG therapy and aspirin are commonly used treatments to reduce inflammation and prevent complications of CALs. However, even with treatment, some children may still develop CALs, highlighting the importance of close monitoring and other potential anti-inflammation agents [18,19]. 

While IVIG treatment significantly reduces the occurrence of coronary artery aneurysm formation, about one-third of KD patients still develop coronary artery dilation during the acute stage. A previous report, based on a serial analysis of coronary artery dilation in 341 patients, showed that 35% of KD patients experienced dilation in the acute stage. One month after disease onset, 17.2% had dilation, 10.2% still had dilation two months after onset, and 4% developed coronary artery lesions (CALs) or aneurysms lasting at least one year. Identifying KD within 5–10 days of disease onset is crucial, as is treating KD with a more precise protocol, especially for children who are IVIG-resistant, in high-risk groups, or have experienced CAL formation. Treating KD patients with the proper dosage of IVIG (a single high dose of 2 g/kg of body weight) over a 10–12 h infusion period, prescribed within the first 5–10 days of the illness, will more effectively reduce CAL formation. IVIG should be administered to KD patients presenting after the 10th day of illness if they have ongoing systemic inflammation, indicated by elevated erythrocyte sedimentation rate (ESR) or C-reactive protein (CRP), persistent fever without another explanation, or aneurysm formation. Patients with KD may be classified into different groups according to disease severity, including KD shock syndrome (KDSS), KD with giant aneurysm formation, KD with aneurysm formation, KD with coronary artery dilation, KD with transient coronary artery dilation, KD with IVIG resistance, and KD without CAL or IVIG resistance. Among these groups, different treatment protocols may be needed, adhering to the principles of precision medicine.

In Suganuma et al.‘s study, a dosage of 1000 μg of LCWE preparation was intraperitoneally injected per mouse to induce aortic–coronary inflammation. In contrast, our dosage was 280 μg per mouse, which is nearly five times lower [20]. The coronary stenosis induced by LCWE is characterized by severe coronary vasculitis and elastin degradation in the histological features of the coronary artery. In contrast, our study found dilatation of the coronary artery using sonography. Stenotic lesions are the most critical coronary arterial lesions in KD because they can induce myocardial ischemia and myocardial infarction, which significantly affect the prognosis. Tsuda et al. reported that the fate of coronary arterial lesions, including the development of coronary stenosis after Kawasaki disease (KD), is related to the degree of coronary arterial dilatation during the acute phase. Therefore, the incidence of subsequent stenosis and the regression of coronary dilatation depend on the coronary arterial diameter during the acute phase in both branch and bifurcation lesions [21]. In summary, coronary artery dilatation is a sign of acute-stage KD, while coronary artery stenosis is a severe complication that can follow dilatation.

CALs in KD are believed to result from inflammation and damage to the coronary artery walls. One proposed mechanism for this damage involves the role of reactive oxygen species (ROS). ROS can contribute to vascular damage through various mechanisms, including the oxidative modification of lipids, proteins, and DNA, as well as the activation of signaling pathways involved in inflammation and cell death. In the context of the coronary arteries or KD, ROS-induced damage to the endothelial cells lining the artery walls may lead to endothelial dysfunction, increased permeability, and the activation of inflammatory processes, ultimately contributing to the development of CALs in KD. Additionally, ROS can interact with other molecules, such as nitric oxide (NO), leading to the formation of reactive nitrogen species (RNS), which can further exacerbate vascular damage and inflammation. The available evidence indicates that vascular endothelial dysfunction is a critical step in the sequence of events leading to the development of cardiovascular lesions in KD [22].

Extensive evidence substantiates the significant involvement of excess ROS production in the development of cardiovascular lesions during KD in children [23,24] and murine models [11,25] of KD vasculitis. Despite the existing anti-inflammatory approaches employed in the treatment of coronary artery abnormalities in KD, their therapeutic efficacy is currently limited, leading to suboptimal treatment outcomes [26,27]. However, there is encouraging news, as the author Kuo HC has reported a remarkable case of a KD patient with a CAA measuring 6.08 mm in diameter and 35 mm in length. This patient experienced regression of the CAA size to within the normal range after four months of inhaling hydrogen gas [28]. The author also reviewed the potential biochemical and chemical aspects of hydrogen gas inhalation in treating KD and COVID-19 [19]. This raises the novel question of whether inhaled hydrogen gas could provide potential clinical benefits as an adjuvant therapeutic approach for KD. In this study, we further confirmed the significant resolution of LCA diameter and Z score after treatment with inhaled hydrogen gas in the KD animal model of LCWE-injected mice. Meanwhile, inhaled hydrogen gas therapy led to a notable decrease in IL-6 expression, which is compatible with the decrease in the formation of CALs in the LCWE murine model of KD vasculitis.

In a multicenter, randomized, double-blind, placebo-controlled trial, it was found that the inhalation of hydrogen may increase 90-day survival without neurological deficits after out-of-hospital cardiac arrest [29]. This novel observation prompts the question of whether inhaled hydrogen gas could provide additional clinical benefits as an adjuvant therapeutic approach for KD. Before conducting large-scale clinical trials in children with KD, it is necessary to assess the safety and efficacy of inhaled hydrogen gas in an animal model of KD, specifically LCWE-induced vasculitis. 

## 5. Conclusions

In the current study, we first demonstrated a significant resolution of LCA diameter and Z score after treatment with inhaled hydrogen gas in an LCWE-induced vasculitis KD animal model. We further found that serum IL-6 expression was higher in mice after LCWE treatment and IL-6 significantly decreased after inhaled hydrogen gas therapy. In conclusion, the inhalation of hydrogen gas was demonstrated to be an effective treatment for coronary dilatation in the KD animal model induced by LCWE.

## Data Availability

The datasets used and/or analyzed during the current study are available from the corresponding author on reasonable request.

## List of Abbreviations

KD	Kawasaki disease
IVIG	intravenous immunoglobulin
CAL	coronary artery lesions
IL-6	interleukin 6
LCWE	*Lactobacillus casei* cell wall extract
ROS	reactive oxygen species
LCA	left coronary artery
